# Striatal and hippocampal contributions to value-based learning in adolescence

**DOI:** 10.1038/s41386-025-02260-7

**Published:** 2025-10-09

**Authors:** Erica Niemiec, Catherine Insel, Juliet Y. Davidow

**Affiliations:** 1 Department of Psychology, Northeastern University, Boston, MA USA; 2 Department of Psychology, Northwestern University, Evanston, IL USA; 3 Institute for Policy Research, Northwestern University, Evanston, IL USA; 4 Institute for Adolescent Mental Health and Well-being, Northwestern University, Evanston, IL USA

**Keywords:** Hippocampus, Human behaviour, Neurological manifestations, Learning and memory, Reward

## Abstract

Adaptive value-based learning is a complex challenge supported by neurobiological systems based in the striatum and hippocampus, with important implications for both everyday behaviors and for mental health. In adults, these systems have been shown to compete, complement, and integrate; less is known about this interplay earlier in development. Here, we discuss representative empirical evidence for the roles of striatum-PFC and hippocampus-PFC systems and their interactions in developing goal-directed behaviors during adolescence. These systems are also implicated in value-based learning alterations in adolescent-emergent mental health disorders. More focus on interactions between value-based learning systems in adolescence, a time of sensitivity to reward and opportunity for mental health interventions, is necessary to support development across healthy and clinical populations. We propose that differential timing of striatum-PFC and hippocampus-PFC network maturation may shape distinct adolescent behavioral phenotypes.

## Striatal and hippocampal contributions to value-based learning in adolescence

Throughout the lifespan, survival in a complex ecosystem requires learning to associate external stimuli with actions that lead to desired outcomes. In value-based learning, the positive or negative reward value of an outcome continually shapes goal-directed action selection based on dynamic expectations of future reward [1]. Value-based learning can result in behavioral patterns that are adaptive, such as making a quip that a potential new friend is likely to find entertaining, or maladaptive, such as the compulsive use of an addictive substance to the detriment of one’s health and relationships. As environments and priorities change with age, value-based learning may manifest differently across contexts, as stimulus salience shifts across development, or with changing magnitude and valence of associated reward values. These changes result in different outcomes for learning, memory, and controlling behavior at different life stages. In this perspective, we focus on the development of striatal and hippocampal systems that support value-based learning in adolescence.

**Fig. 1:**
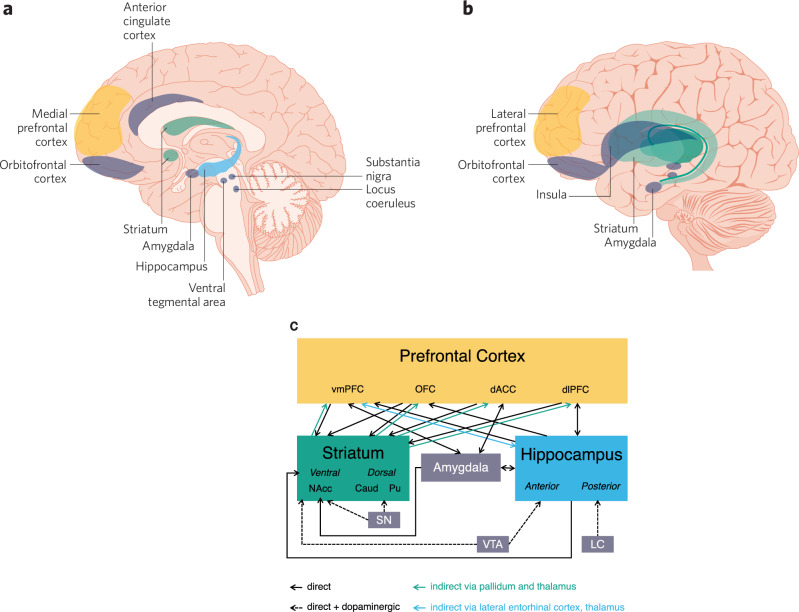
Brain regions supporting value-based learning. **a** Medial and **b** lateral views of regions involved in value-based learning. Focal regions for this perspective are shown in green (striatum), blue (hippocampus), and yellow (prefrontal cortex); other related regions are shown in gray. **c** Connections in circuits involved in value-based learning. vmPFC ventromedial prefrontal cortex, OFC orbitofrontal cortex, dACC dorsal anterior cingulate cortex, dlPFC dorsolateral prefrontal cortex, NAcc nucleus accumbens, Caud caudate, Pu putamen, SN substantia nigra, VTA ventral tegmental area, LC locus coeruleus. Black arrows indicate direct connections, dotted-line arrows indicate dopaminergic connections, green arrows indicate indirect connections via pallidum and thalamus (not pictured), and blue arrows indicate indirect connections via lateral entorhinal cortex or thalamus (not pictured).

Learning to adaptively perform reward-eliciting behaviors is therefore a complex challenge requiring contributions from multiple neural systems (Fig. 1). Simple stimulus-action-outcome associations in value-based learning rely on the striatum, a hub for dopaminergic neurocircuitry; converging inputs to the striatum from the amygdala, hippocampus, and cortical regions guide complex reward representation and action selection [1]. Findings also support a major role for the hippocampus, which receives dopamine projections from the ventral tegmental area and locus coeruleus [2] along with indirect, and sparse direct, connections to the striatum [3], in value-based learning [4]. Additionally, executive functions and other aspects of higher-order cognition supported by prefrontal regions, including medial and ventromedial prefrontal cortex, orbitofrontal cortex, anterior cingulate cortex, and dorsolateral prefrontal cortex, contribute to forms of value-based learning that require representations of environmental structures or integration of multiple forms of feedback [1]. These behaviors, the neural systems that support them, and their interactions continue to develop throughout adolescence. Learning-related developmental plasticity during this period, therefore, confers both vulnerability for mental health and opportunity for intervention. Growing evidence has focused on the role of the striatum in guiding adolescent learning, yet relatively few empirical studies have examined the complex interplay between multiple learning systems during adolescent development. Here, we propose that value-based learning trajectories can be interpreted through a framework in which different forms of cross-system interaction develop during adolescence, and this may inform neural and behavioral alterations in psychopathology.

In human adults, striatal and hippocampal systems may compete, complement, or integrate during learning. That is, they may be dissociable, functioning independently or interfering with one another (compete); act in parallel to support the same behaviors in different contexts (complement); or one system may modify the other’s operation (integrate). Evidence of hippocampal-striatal competition has been shown by a double dissociation in which declarative memory is impaired in amnesic patients with hippocampal damage, while probabilistic learning is impaired in Parkinson’s patients with striatal damage [5]. However, there is also evidence for a hippocampal role in probabilistic learning, complementing the function of the striatum [6, 7]. Additionally, hippocampal-driven generalization has been shown to support reward learning by increasing preference for previously non-rewarded events when paired with rewarded events [8], and reward enhances memory for salient events to improve predictions via connectivity between the ventral tegmental area, cortex, and hippocampus [9]. Thus, striatal and hippocampal learning systems may also integrate to support goal-driven behavior. In what contexts do each of these learning functions predominate, and how do they mature? Adolescence is a developmental period of special interest for exploring these questions, in part because ongoing maturation of circuits connecting learning systems with prefrontal cortical regions presents opportunities for understanding mechanisms underlying mental health challenges [10].

The boundaries of adolescence are debated, differing across species and, within humans, across historical and cultural contexts. Here, we define adolescence based on features shared across mammalian species: a developmental transition from caretaker dependence to relative independence, within which pubertal maturation occurs [11]. The striatum, hippocampus, and their connections with prefrontal cortical regions undergo protracted structural and functional development across adolescence [1]. Adolescence is also a period of increasing autonomy and sensitivity to reward due in part to increased dopamine signaling, making value-based learning especially salient and agentic [12]. Finally, and perhaps most crucially for this special issue, adolescence has been increasingly recognized as a window of risk and opportunity for intervention in mental health disorders. Alterations in value-based learning manifest in substance use disorders, depression, eating disorders, ADHD, and schizophrenia, many of which typically onset during adolescence or young adulthood [10].

While adult work has established the complex interplay between these systems, developmental work has typically tested striatal and hippocampal systems in isolation. Moreover, research on adolescence has largely focused on how hippocampal and striatal circuitry separately interact with prefrontal cortical regions. Here, we provide a brief overview of each system’s development, including contributions of cognitive functions supported by interactions with regions of the prefrontal cortex. Because developmental literature in this area is sparse, we then focus on specific studies offering benchmark evidence of the role of prefrontal cortical maturation in value-based learning within each system and emerging evidence for between-system interactions in adolescence. We propose new hypotheses about how the development timing of hippocampal-prefrontal and striatal-prefrontal network maturation may shape distinct behavioral repertoires during adolescence. Finally, we discuss the implications of altered value-based learning systems for adolescent mental health. A deeper understanding of these systems and their interactions will aid in the more precise identification of risks, interventions, and opportunities for resilience in adolescence and throughout life.

## Striatum-PFC and hippocampus-PFC systems in development

From its classic associations with motor control, understanding of the striatum has evolved to incorporate a role as an integration hub for a wide range of functions involved in goal-directed behavior, including action selection, reward anticipation, and learning from feedback [3]. Multiple connections with limbic regions, primarily in the ventral striatum (i.e., nucleus accumbens), [3] and overlapping inputs supporting executive and motor functions via corticostriatal loops primarily to the dorsal striatum (caudate and putamen) [13, 14], make the striatum ideally positioned in the brain for this integration. The development of corticostriatal circuits follows a medial to lateral gradient: the integrity of projections from cortical regions involved in emotional and reward processing (including orbitofrontal cortex) reduces through adolescence into adulthood, in contrast to projections from regions involved in regulatory control of cognitive, emotional, and behavioral processes (including ventrolateral and dorsolateral prefrontal cortex) [14]. From infancy (e.g., 8 months) [15] and across development, reward signals drive learning and motivated behavior; however, different maturational rates of striatal connections influence changes in reward-motivated behavior. Mesolimbic dopaminergic connections between the ventral tegmental area and ventral striatum mature before cortical connections, conferring heightened sensitivity to reward and perhaps can contribute to developing psychopathology in adolescence [11, 14]. For example, heightened reward sensitivity may strengthen reinforcement in substance use or increase vulnerability to environmental stressors in mood disorders; plasticity in maturing corticostriatal circuits also allows for these networks to be reshaped by substance use or psychopathological behavior during adolescence.

While converging evidence posits that adolescents exhibit heightened reward sensitivity in the striatum, developmental trajectories vary depending on cognitive tasks, motivational framing (gain versus loss, type of reward, receiving or omitting a reward), and age sampling [16–21]. For example, there is evidence for an adolescent peak in reward sensitivity concurrent with a peak in ventral striatum activation [18], similar levels of ventral striatum activation but differences in orbitofrontal cortex activation for adolescents and adults following reward [20], and different developmental patterns of activation for tracking magnitude of rewards gained (in caudate) or lost (in insula) [21]. Variance may indicate changes in how memories of previous experiences contribute to reward valuation, how reward-related events are remembered, and how executive functions are involved in modulating what is attended to and therefore learned or remembered. To explain this variance, it may be helpful to consider the roles of multiple neural systems supporting these functions in value-based learning, especially those involving prefrontal cortical regions, in adolescence [1]. Refinement of corticostriatal connectivity from adolescence into adulthood may support an increased ability to appropriately calibrate effort and select actions in value-based learning [22, 23]. Various cortical subregions perform specific roles within this broader function. Moving medially to laterally, the orbitofrontal cortex links sensory information to rewarding outcomes; the anterior cingulate cortex is involved in conflict monitoring and action selection. The ventromedial prefrontal cortex supports flexible valuation of stimuli and representation of future rewards, the ventrolateral prefrontal cortex guides response inhibition and flexible updating, and the dorsolateral prefrontal cortex facilitates strategic planning and context maintenance [3]. As dopaminergic pathways connecting subcortical regions with prefrontal cortical regions mature, these cortical functions are increasingly involved in value-based learning.

The hippocampus is also a critical structure that develops during adolescence to support the maturation of value-based learning. Famously known as a center for memory, the hippocampus supports multiple functions, including episodic memory, statistical learning, associative memory, model-based learning, temporal representation, and future thinking. There is evidence for a dorsal/posterior to ventral/anterior gradient in hippocampal functions, informed by a gradient of connectivity with subcortical and cortical regions. The ventral/anterior hippocampus is most strongly connected to the ventral striatum, amygdala, and regions involved in reward and emotional processing, supporting a role for this subregion in motivational and affective learning, while the dorsal/posterior hippocampus is most strongly linked with regions involved in sensory and spatial processing, supporting a role in recalling details [24]. Furthermore, associative memories that flexibly generalize over previous experiences to inform learning in novel situations are supported by the anterior hippocampus and its integration with ventromedial regions in the prefrontal cortex [2]. While simple recall develops before middle childhood (e.g., ages 6–8 years), performance on episodic and complex memory tasks is slower to mature (see ref. [25] for review). This protracted developmental trajectory is informed by increased contributions of the prefrontal and parietal cortex to learning and memory challenges requiring cognitive control and attentional modulation [25], including mature value-based learning. The maturation of dopaminergic circuitry may support developing hippocampocortical connections and contributions of prefrontal cortical regions to hippocampally-supported learning and memory [2].

## Multiple systems support flexible value-based learning in adolescence

Taken separately, developing striatum-PFC and hippocampus-PFC systems support increasingly complex, goal-directed learning and memory behaviors. However, minimal work in humans has examined how interactions between these systems influence learning during adolescence [2]. We propose that the developmental tempo of maturation in these systems influences the ways in which adolescents learn from reward and punishment. We might then expect that in adolescence, maturation of between-systems connections supported by heightened dopaminergic function could further support the emergence of complex, situational interactions (competition, coordination, or integration) evident in adults. The nature of between-system interactions in adolescence could provide insights into neurobiological development, and understanding the development of these dynamic circuits can also inform our understanding of basic brain function. For example, between-system competition may predominate early in life. However, with age, maturing between-system connections could increasingly support complementary and integrative functions across adolescence into adulthood. It is also possible that earlier maturation of mesolimbic reward and corticostriatal socioemotional processing circuits within the striatum-PFC system may inform the development of the hippocampus-PFC system, or that developmental shifts in either or both systems’ function could contribute to emerging psychopathology.

**Fig. 2:**
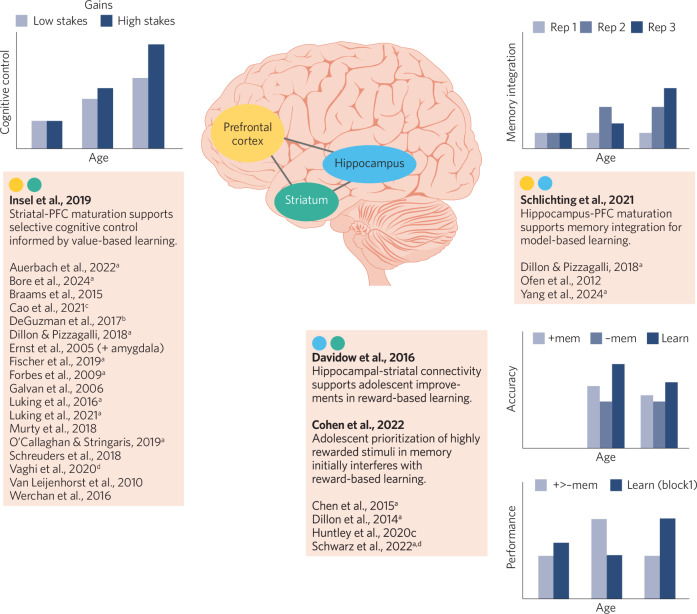
Striatum-PFC and hippocampus-PFC systems and their interactions across development. Boxes show selections from the literature that discuss the development of value-based learning and memory in the striatum and striatum-PFC system (box labeled yellow and green), the hippocampus, medial temporal lobe, and hippocampus-PFC system (box labeled yellow and blue), and interactions between striatum-PFC and hippocampus-PFC systems (box labeled blue and green). Bar graphs display simplified illustrations of key results for each of the four focus papers discussed in the main text (authors shown in bold). Papers with an alphabetical superscript discuss clinical implications for depression^a^, anorexia nervosa^b^, substance use disorder^c^, other disorders^d^.

While these questions remain unanswered, insights can be drawn from comparing emerging evidence in adolescent value-based learning tested separately within striatal and hippocampal systems. Below, we narratively review empirical studies that find contributions of striatum, hippocampus, and prefrontal regions in the development of value-based learning (Fig. 2). Frontostriatal circuits are the best established in the literature for their critical role in value learning, in development, and in adult and animal models. Far less evidence in development implicates hippocampus-PFC and hippocampus-striatum circuitry, despite a growing literature in adult and animal models. To promote additional developmental work to fill this gap, we discuss two studies highlighting different forms of between-systems interactions in adolescence. These studies underscore that identifying contexts in which striatum-PFC and hippocampus-PFC systems compete, coordinate, or integrate can clarify conflicting developmental trajectories of value-based learning.

### Striatum-PFC

Frontostriatal systems play a central role in both reward learning and cognitive control, and adult research has found that interactions between the striatum and PFC influence when and how previously learned reward associations shape future cognitive control deployment [26]. Evidence from developmental research has extended this understanding of frontostriatal interactions by revealing that, during adolescence, striatum-PFC connectivity strengthens with age and supports the ability to carry forward previously learned reward associations into new contexts to guide adaptive behavior. Specifically, prior work trained monetary value associations during a reinforcement learning task and then tested how these previously learned cues influenced subsequent cognitive control during an inhibitory control task, when participants had to inhibit responses to previously incentivized cues. Older adolescents, but not younger adolescents, improved cognitive control performance when inhibiting responses to cues that had been previously paired with high-value rewards [27]. Importantly, these differences in behavior could not be attributed solely to changing motivation to earn more rewards, since the inhibitory control task was not incentivized, and a control analysis of subjective preferences for monetary rewards showed no effect of age. These behavioral changes were paralleled by age-related increases in frontostriatal recruitment. Older adolescents exhibited more activity in the ventrolateral prefrontal cortex and caudate when inhibiting responses to high-value cues, and functional connectivity between the caudate and dorsolateral and ventromedial prefrontal cortex increased with age [27]. These results highlight the role of frontostriatal circuit maturation in supporting an emerging ability to integrate learned value associations with cognitive control demands during adolescence. These findings also align with research demonstrating increased cortical involvement with age as a mediator in reduced coactivation between the nucleus accumbens and ventral tegmental area in motivational contexts [23]. However, this study did not test whether maturing memory systems contributed to the transfer of value representations across contexts—this presents an opportunity for future work examining the contribution of multiple neural systems to value-based cognition.

### Hippocampus-PFC

Hippocampus-PFC circuits are also essential for flexible goal-directed behavior, and these connections support goal-directed planning and model-based learning. In adults, coordinated recruitment of the hippocampus and PFC guides the integration of past experiences into cognitive models of environmental states, which can then inform behavior in novel situations[8]. There is emerging evidence that these integration mechanisms mature during adolescence. For example, prior work using a task in which participants learned to infer a novel relationship from overlapping pairs of stimuli, followed by a memory test for the paired items, found age-related increases in reactivation of related memories using a multivariate fMRI classification analysis [28]. Adolescents displayed reactivation only on the second repetition, consistent with memory differentiation, while adults maintained reactivation across repetitions, consistent with integration. Reactivation was associated with activity in the parahippocampal cortex, parietal cortex, and inferior frontal gyrus, with age-related changes in the relationship between reactivation and engagement of the parietal cortex and inferior frontal gyrus [28]. These findings suggest that improvements in applying memory integration to support learning rely on developmental changes in the prefrontal cortex and cortical interactions with memory-supporting regions in the medial temporal lobe. This work also demonstrates the presence of different memory mechanisms at different developmental stages, perhaps in response to shifting developmental demands, emphasizing the likelihood of nonlinear developmental patterns in the hippocampus-PFC system. While this study describes the development of a key mechanism related to value-based learning, it did not examine how memory reactivation might be modulated by changing value representations. Future work should explicitly test the connection between memory reactivation and its applications in value-based learning.

### Hippocampus-striatum

Striatum-PFC and hippocampus-PFC systems also interact in complex ways to support goal-directed behavior. Heightened sensitivity to reward in adolescence has been proposed to facilitate adaptive exploration as independence increases [1]. If so, integration of striatum-PFC and hippocampus-PFC systems could support memory prioritization for rewarding stimuli, improving learning in high-reward contexts. This possibility is suggested by results from a reinforcement learning task in which adolescents demonstrated better learning than adults. Better learning was associated with stronger reward-related activation of the hippocampus and stronger functional connectivity between the hippocampus and the striatum [29]. In the context of adolescent neurobiology, control mechanisms that could arbitrate in competitive or complementary functions of the striatum and hippocampus are supported by still-immature connections between these regions and the prefrontal cortex [12]. Increased hippocampal-striatal connectivity may then facilitate cooperative integration in adolescence. To investigate this possibility, future work should explore the role of executive functions supported by prefrontal cortical regions in arbitrating hippocampal–striatal interactions and test whether prefrontal regions regulate hippocampal–striatal connectivity.

As hippocampus-PFC integration supports increased memory differentiation in adolescence, reward-related memory enhancements may also interfere with value-based learning, particularly if remembered information is no longer relevant in a new context. In a probabilistic learning task following memory encoding, adolescents demonstrated stronger general source memory prioritization of highly rewarded stimuli than children or adults, and early learning performance for previously highly rewarded stimuli was impaired [30]. Importantly, however, this decrement during early learning was ameliorated as learning continued, and learned reward values did not continue to bias choices following learning. These results suggest that increased orientation towards reward in adolescence may sometimes lead to “false starts”, which are generally corrected by normative learning processes as experience accumulates. If not adjusted, however, impaired learning related to heightened memory for affective stimuli could increase mental health risk during adolescence. Although in this study, individual differences in reward-related memory prioritization had less influence on learning in the adolescent group than in children or adults, it remains important to consider the role of variability in reward-related mechanisms for the development of mental health disorders. Future work should identify contexts in which competitive, coordinative, or integrative functions of the hippocampal and striatal system predominate and how this contributes to individual differences in goal-directed behavior.

## Clinical implications and future directions

The majority of mental disorders emerge before age 25, with many peaking during adolescence [10]. Clarifying the neurodevelopmental mechanisms that confer risk for mental health disorders is therefore critical for early identification and developmentally appropriate interventions. Alterations in value-based learning and memory have been identified across disorders that commonly emerge in adolescence, including eating disorders, depression and anxiety, and substance use disorders [31], which we focus on here, though these processes may be important to consider in other disorders such as psychosis. The transdiagnostic nature of these alterations makes them an especially promising focus for future study, as a clearer picture of value-based learning and memory mechanisms has the potential for broad impact on mental health.

One pattern of transdiagnostic alterations in value-based learning involves reduced sensitivity to and impaired learning from rewards, along with structural and functional alterations in striatal circuits. For example, reduced striatal volume [32] and activation have been demonstrated in reward anticipation and response in adolescents with depressive symptoms [33]. Compensatory cognitive control mechanisms supported by prefrontal cortical regions have been related to depression resilience [33], highlighting the potential of neuroimaging and predictive modeling approaches to identify neurobiological markers, as well as the need to consider the interplay between executive functions and value-based learning in frontoparietal networks. Altered reward learning has also been observed in adolescents with eating disorders [34]. In anorexia nervosa, adolescents have shown heightened functional activation for reward prediction error in the striatum and insula [35], suggesting striatal reward prediction error as a neurobiological marker of disorder severity.

A second pattern, though less broadly demonstrated across disorders, involves memory-related changes paralleled by hippocampal alterations. Behavioral changes in adolescent depression involve alterations in reward-related memory alongside hippocampal alterations [36]. These alterations suggest potential focal points for intervention: hippocampally-targeted transcranial magnetic stimulation has shown preliminary promise in reducing symptoms of depression and bipolar disorder during adolescence [37]. Characterizing the effects of such interventions across hippocampus-PFC and hippocampal-striatal circuits is essential to maximize their therapeutic potential.

Finally, there is emerging evidence for atypical hippocampal-striatal connectivity in adolescent-emergent mental health disorders, suggesting alterations in the integration of reward- and memory-related systems. For example, altered functional connectivity between the ventral striatum and medial temporal gyrus during reward processing has been associated with increases in adolescent alcohol use [38]. Similarly, higher resting-state functional connectivity between the ventral striatum and hippocampus has been linked to longitudinal increases in adolescent substance use [39]. This implies that adolescence may be a time not only of sensitivity to reward, but sensitivity to reward-related modulations of memory, and that this sensitivity confers vulnerability for substance use disorders. However, sensitivity may also confer opportunity. Striatal and hippocampal system interactions during adolescence have been linked to heightened music-related memory and reward valuation, suggesting the potential for interventions targeting these circuits to benefit adolescent mental health [40]. Thus, leveraging multiple learning systems in interventions may be particularly beneficial during adolescence.

Development of interventions will require additional research focused on clarifying the nature of interactions between neural systems, including the role of cortical maturation in cross-system integration, and how these interactions are informed by specific contexts in development. Longitudinal neuroimaging and computational modeling approaches will be especially valuable in characterizing age- and context-related changes in value-based learning [31, 41]. In addition, multivariate neuroimaging and precision functional mapping can aid in unraveling the complexities of within- and between-system interactions, especially because networks and their component connections develop at different rates within and across individuals. Approaches that bridge multiple levels of neural system organization and behavior can also be beneficial. For example, research in pre-clinical animal models and post-mortem human studies indicate a peak in dopamine signaling during adolescence [2]; neuroimaging methods that noninvasively measure longitudinal and cross-sectional change in brain-tissue iron accumulation, as an index of dopaminergic processes, along with measures of structural and functional network connectivity, could provide more information about the development of dopamine’s role in integrating striatal, hippocampal, and prefrontal cortical circuits [42]. Advances in neurobiological specificity can lay the foundation for new, more focused clinical interventions tailored to neural system development. At developmental stages when complementary and integrative cross-system interactions are maturing, treatments could leverage one system to support another that is typically altered in psychopathology. Protracted maturation of prefrontal cortical connections with both systems confers opportunity for these circuits to be shaped by early treatment, if markers of risk and resilience and their manifestation within the developmental timeline are specified.

In  this Perspective, we have aimed to highlight the challenges and opportunities involved in characterizing striatum-PFC and hippocampus-PFC value-based learning systems in development. Goal-directed behavior is complex, requiring contributions from many processes with distinct developmental patterns and shifting interactions between them. Few studies have considered the nature of developing striatal-hippocampal interactions, despite the role of dopamine in both striatal [42] and hippocampal [2] systems, and overlapping anatomical connections with the prefrontal cortex. Future work should therefore experimentally evaluate the changing contributions of multiple learning systems across development, especially in adolescence, a time of rapid cortical maturation and refinement of cortico-subcortical connections supported by enhanced dopaminergic function [2]. Because existing literature has highlighted the importance of contextual elements in shaping trajectories of value-based learning, it will be important to consider how these contexts, including cultural elements, contribute to developing value-based learning systems. Investigating the developmental trajectories of reward-related learning and memory functions may support earlier diagnosis of emerging mental disorders and identify optimal methods and timing for intervention. Patterns of alterations across mental health disorders and adolescent-timed maturational changes in typical development suggest that value-based learning systems are promising focal points for supporting adolescent mental health.
